# TAK-875 Mitigates *β*-Cell Lipotoxicity-Induced Metaflammation Damage through Inhibiting the TLR4-NF-*κ*B Pathway

**DOI:** 10.1155/2019/5487962

**Published:** 2019-12-17

**Authors:** Xide Chen, Yuanli Yan, Zhiyan Weng, Chao Chen, Miaoru Lv, Qingwen Lin, Qiuxia Du, Ximei Shen, Liyong Yang

**Affiliations:** ^1^Endocrinology Department, The First Affiliated Hospital of Fujian Medical University, Fuzhou, 350005 Fujian, China; ^2^Diabetes Research Institute of Fujian Province, Fuzhou, 350005 Fujian, China

## Abstract

Metabolic inflammatory damage, characterized by Toll-like receptor 4 (TLR4) signaling activation, is a major mechanism underlying lipotoxicity-induced *β*-cell damage. The present study is aimed at determining whether G protein-coupled receptor 4 (GPR40) agonist can improve *β*-cell lipotoxicity-induced damage by inhibiting the TLR4-NF-*κ*B pathway. Lipotoxicity, inflammation-damaged *β*-cells, obese SD, and TLR4^KO^ rat models were used in the study. In vitro, TAK-875 inhibited the lipotoxicity- and LPS-induced *β*-cell apoptosis in a concentration-dependent manner, improved the insulin secretion, and inhibited the expression of TLR4 and NF-*κ*B subunit P65. Besides, silencing of TLR4 expression enhanced the protective effects of TAK-875, while TLR4 overexpression attenuated this protective effect. Activation of TLR4 or NF-*κ*B attenuated the antagonism of TAK-875 on PA-induced damage. Moreover, the above process of TAK-875 was partially independent of GPR40 expression. TAK-875 reduced the body weight and inflammatory factors, rebalanced the number and distribution of *α* or *β*-cells, inhibited the apoptosis of islet cells, and inhibited the expression of TLR4 and NF-*κ*B subunit P65 in obese rats. Further knockout of the rat TLR4 gene delayed the damage induced by the high-fat diet and synergy with the action of TAK-875. These data suggest that GPR40 agonists antagonized the lipotoxicity *β*-cell damage by inhibiting the TLR4-NF-*κ*B pathway.

## 1. Introduction

Long-term high-fat diet could cause obesity and insulin resistance [1–4], which is an important cause of diabetes [5]. An increasing number of studies demonstrated that metabolic inflammatory response is crucial for lipotoxicity to play a role in *β*-cell damage [6–9]. Our previous studies also showed that lipotoxicity initiates the inflammatory reactions in *β*-cells, leading to *β*-cell insulin secretion disorder, inhibition of insulin secretion-related gene expression, cyclin expression disorder, and induction of *β*-cell apoptosis [10]. Presently, several studies have confirmed that the inhibition of metabolic inflammation significantly improves the lipotoxicity damage of *β*-cells [11]; however, a specific intervention has not yet been described, thereby necessitating further exploration of the targeted therapeutic measures.

G protein-coupled receptor 40 (GPR40) is a medium- and long-chain FFA receptor that amplifies the glucose-stimulated insulin secretion (GSIS) response [12, 13]. In addition, we also confirmed that the activation of GPR40 ameliorates the *β*-cell insulin secretion damage caused by lipotoxicity [14] and mediates the pioglitazone-antagonizing lipotoxic apoptosis of *β*-cells [15]. Recent studies demonstrated that the activation of GPR40 reverses the inflammatory cell-induced apoptosis in *β*-cells [16], as well as ameliorates insulin resistance [17]. Furthermore, GPR40 and GPR120 synergize in the hypothalamus to regulate the inflammation associated with obesity [18]. However, whether GPR40 exerts a protective effect on *β*-cell lipotoxicity by antagonizing the metabolic inflammation is yet to be substantiated.

Metabolic inflammation is an important pathophysiology of obesity-induced diabetes [19]. Toll-like receptor 4 (TLR4) is a key factor in the innate immune activation of the inflammatory signaling pathway [20, 21], which plays a critical role in the pathogenesis of type 2 diabetes metabolic inflammation [22]. Previous studies have shown that TLR4 overactivation leads to insulin resistance [23] and causes islet *β*-cell inflammatory infiltration [24] and islet secretion dysfunction [25]. Moreover, lipotoxicity directly activates the TLR4-JNK pathway cascade in islet *β*-cells and induces *β*-cell insulin secretion disorders [15, 26]. Some of the recent studies have shown that TLR4 knockout (KO) can antagonize the damage caused by a high-fat diet (HFD) and aging by reducing the expression of insulin-sensitive tissues and islet inflammation products [27]. In addition, pioglitazone, a nonspecific agonist of GPR40, ameliorates the lipotoxic *β*-cell damage by inhibiting the TLR4 signaling [15]. However, whether GPR40 agonists improve the metabolic inflammatory damage of *β*-cells by inhibiting the TLR4 signaling pathway has not yet been reported.

In summary, the present study is aimed at investigating the relationship between the protective effect of GPR40 on lipotoxicity-induced metabolic inflammatory injury and the relationship with the TLR4-NF-*κ*B pathway by using a *β*-cell strain, HFD-induced obese rats, and TLR4^KO^ rats as experimental tools.

## 2. Methods and Materials

### 2.1. Cell Culture

*β*TC6 is mouse islet cell tumor cell line purchased from the ATCC (USA). Cells were cultured using a complete medium (containing Dulbecco's modified Eagle's medium (DMEM, 4.5 g/L glucose), 10% fetal bovine serum (FBS), 2 mmol/L L-glutamine, 10 mmol/L HEPES, 100 U/mL penicillin, and 100 *μ*g/mL streptomycin) at 37°C in a 5% CO_2_ incubator. All reagents were purchased from Gibco (USA). The cells at passages 20–25 were used for subsequent experiments.

### 2.2. Construction and Transfection of TLR4 siRNA and TLR4 Overexpression

TLR4 siRNA silencing was conducted as described previously [15]. The GV492 vector to TLR4 cDNA of mice was provided by Shanghai GeneChem Co., Ltd. (Shanghai, China). Primers used for TLR4 cDNA synthesis were as follows: TLR4(24379-1)-P1, AGGTCGACTCTAGAGGATCCCGCCACCATGATGCCTC CCTGGCTCCTGGCTAGG; and TLR4(24379-1)-P2, TCCTTGTAGTCCATACCGGTCCAAGTTGCCGTTTCTTGTTCTTC. TLR4 cDNA lentiviral vector was diluted to 1 × 10^8^ IU/mL. After multiplicity of infection (MOI) 50, we diluted it into HDMEM+10% FBS with 2 mmol/L L-glutamine. After 10–12 h incubation, the transfected medium (enhanced infection solution+HDMEM+10% FBS+2 mmol/L L-glutamine) was replaced by a fresh complete medium, and the cells were incubated for 72 h. The expression of TLR4 protein was detected by western blot.

### 2.3. GPR40 Small Interfering (si) RNA and Adenovirus Generation

The specific experimental process was based on our previous methods [15].

### 2.4. Cell Interference

#### 2.4.1. PA Interference

The preparation of palmitic acid (PA) is referring to the method described by Ke et al. [28]. *β*TC6 cells were pretreated with 0.5 mmol/L PA for 24 h to establish a lipotoxicity model as described previously [15].

#### 2.4.2. TAK-875 Interference

*β*TC6 cells were intervened with 0.5 mmol/L PA and different concentrations of GPR40 agonist (TAK-875) for 72 h. Each group was subjected to the treatment three times (0.5 mmol/L PA+25 nmol/L TAK-875, 0.5 mmol/L PA+50 nmol/L TAK-875, and 0.5 mmol/L PA+100 nmol/L TAK-875; TAK-875 was solubilized in 0.01% DMSO (*v*/*v*); Selleck), independently.

#### 2.4.3. LPS Interference

*β*TC6 cells were pretreated with 1.0 *μ*g/mL of TLR4 signaling agonist lipopolysaccharide (LPS, solubilized in 0.01% DMSO (*v*/*v*), Sigma-Aldrich) for 4 h before exposure to 0.5 mmol/L PA and 100 nmol/L TAK-875 for 72 h. The cells exposed to a LPS medium or a complete medium were used as controls.

#### 2.4.4. TNF-*α* Interference

*β*TC6 cells were exposed to 10 ng/mL NF-*κ*B agonist Tumor Necrosis Factor-*α* (TNF-*α*, solubilized in 0.01% DMSO (*v*/*v*); Sigma-Aldrich) in a complete medium for 4 h before exposure to 0.5 mmol/L PA and 100 nmol/L TAK-875 for 72 h. The cells exposed to a TNF-*α* medium or a complete medium were used as controls.

### 2.5. Animal Experiment

SPF SD male rats (Fujian Medical University Animal Center) and TLR4 gene knockout (KO) SD male rats (GenePharma Co., Ltd) were 5-6-week-old, weighing 220 ± 18 g. The animals were fed adaptively for 1 week and then randomly divided into general feeding and high-fat diet-fed groups [6]. After 16 weeks, the general feeding group was divided into the blank control, TLR4^KO^, and TLR4^KO^+TAK-875 groups. The HFD group was divided into HFD, HFD+TLR4^KO^, and HFD+TLR4^KO^+TAK-875 groups (TAK-875 10 mg/kg/day, gavage, *n* = 10); the TAK-875 intervention was 11 weeks. The obesity in the rats was defined as an average weight gain of 15% in the normal group [29]. The weight and length were recorded every 2 weeks during the study. The study was approved by the Biomedical Research Ethics Committee of the First Affiliated Hospital of Fujian Medical University.

### 2.6. Biochemical Indexes and Inflammatory Markers of Rat Serum

Rats were anesthetized by intraperitoneal injection of pentobarbital (60 mg/kg body weight). Blood samples were collected by the abdominal aortic method. The inflammatory factors (IL-1, IL-6, and TNF-*α*) were detected by enzyme-linked immunosorbent assay (ELISA). The insulin level was detected by radioimmunoassay. The fasting blood glucose (FBG) was detected by glucose oxidase. Triglycerides (TG), total cholesterol (TC), and low-density lipoprotein (LDL) were detected by enzyme colorimetry. The homeostasis model assessment of *β*-cell function (HOMA-B) is a method for assessing insulin resistance from basal glucose and insulin concentrations. The formula is as follows: HOMA‐B = 360 × fasting insulin (U/mL)/[fasting blood glucose (mg/dL)–63] [30].

### 2.7. Western Blotting

All *β*TC6 cells and rat islet *β*-cells were collected. The islets were isolated from the pancreas of SD rats [31]. SDS-PAGE, transfer to PVDF membrane, incubation with antibodies, and image analysis were carried out as described previously [15].

### 2.8. Immunofluorescence

Paraffin-embedded sections of pancreatic tissue were dewaxed, hydrated, and pretreated with the heat-induced antigen retrieval technique. Each sample was subjected to enzyme inactivation with 3% H_2_O_2_-methanol solution for 10 min and blocked with goat serum for 20 min. Then, each sample was incubated with 50–100 *μ*L of 1 : 50 diluted primary antibody (anti-insulin and antiglucagon) in a humid chamber for 2 h at 37°C, followed by addition of 50–100 *μ*L 1 : 50 diluted second antibody FITC/TRITC and incubation in the dark for 1 h at 37°C. Each slice was stained with DAPI in the dark place at room temperature for 5 min and mounted with an antifade mounting medium. The expression of the protein was observed and photographed by fluorescence microscopy.

### 2.9. Apoptosis Analysis by TUNEL

#### 2.9.1. Detection of *β*TC6 Cells

TUNEL was performed using a DeadEnd™ Fluorometric TUNEL System (Promega, Madison, WI, USA), as described previously [15].

#### 2.9.2. Detection of Animal Pancreatic Tissue

Paraffin sections of pancreatic tissue were dewaxed, hydrated, and washed two times with xylene for 5 min each time, followed by different concentrations of ethanol (100, 95, 90, 80, and 70%) for 3 min each. The samples were treated with 1% Triton X-100 and 3% H_2_O_2_-methanol solution for 15 min each time. Then, proteinase K was added dropwise at 37°C for 30 min. Subsequently, streptavidin-FITC-labeled working solution was added to each section, and the reaction was conducted in a humidified chamber at 37°C for 1 h in the dark. Each slice was then subjected to DAPI staining for 5 min at room temperature in the dark light. The apoptotic cells were observed and photographed by fluorescence microscopy.

### 2.10. Hematoxylin-Eosin (HE) Staining

The pancreatic tissue sections were fixed in 4% paraformaldehyde for 24 h, followed by dehydration and transparent and embedded into paraffin in a 60°C oven overnight. Then, the slices were dewaxed in water and 3% H_2_O_2_-methanol solution at room temperature for 20 min, followed by washes in double distilled water wash for 5 min each (3 times). Subsequently, the sections were stained with Harris's hematoxylin solution for 12 min, subjected to 75% hydrochloric acid-ethanol differentiation for 30 s, washed with water blue, diluted in distilled water, and dehydrated in 95% ethanol 1 min each (2 times), 100% ethanol 1 min each (2 times), xylene-carbonic acid solution (3 : 1) for 1 min, and xylene for 1 min each (2 times). Finally, after drying, the slide was sealed with a coverslip, and the islets were observed under a light microscope.

### 2.11. Glucose-Stimulated Insulin Secretion (GSIS)

GSIS was assessed as described previously [15]. Insulin was determined by an ELISA Kit (Cusabio).

### 2.12. Statistical Analysis

SPSS 20.0 was used for statistical analysis. Data were represented by mean ± standard deviation (*x* ± *s*). The comparisons between the groups were performed using ANOVA. The LSD test was used for comparison between two groups. *P* < 0.05 was considered as a statistically significant difference.

## 3. Results

### 3.1. TAK-875 Improved PA or LPS-Induced *β*-Cell Damage in a Dose-Dependent Manner

**Figure 1:**
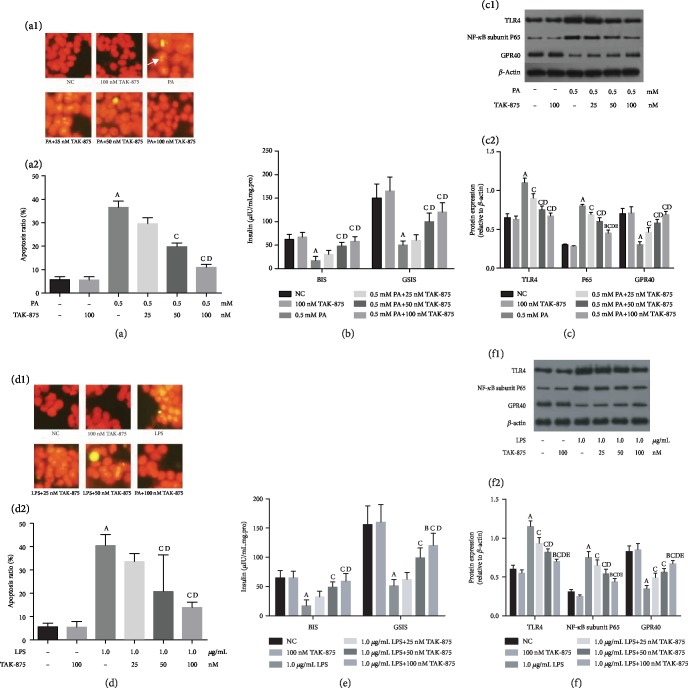
TAK-875 improved PA or LPS-induced *β*-cell damage in a dose-dependent manner. (a–c) TAK-875 improves PA-induced apoptosis (the white arrow indicates the apoptotic cells) (a), insulin secretion disorder (b), and TLR4 and NF-*κ*B subunit P65 expression (c). (d–f) TAK-875 improves LPS-induced apoptosis (d), insulin secretion disorder (e), and TLR4 and NF-*κ*B subunit P65 expression (f). ^a^*P* < 0.05 vs. NC group (without PA and TAK-875), ^b^*P* < 0.05 vs. 100 nmol/L TAK-875 group, ^c^*P* < 0.05 vs. 0.5 mmol/L PA group, ^d^*P* < 0.05 vs. 0.5 mmol/L PA+25 nmol/L TAK-875 group, and ^e^*P* < 0.05 vs. 0.5 mmol/L PA+50 nmol/L TAK-875 group.

To observe the effect of TAK-875 on the lipotoxicity-injured *β*-cells, the cells were incubated with different concentrations of TAK-875. We observed that TAK-875 decreased the apoptosis of islet *β*-cells in a concentration-dependent manner (Figure 1(a)), increased the insulin secretion, which includes BIS, GSIS (Figure 1(b)), and GPR40 expression, and decreased the TLR4 and NF-*κ*B subunit P65 expression (Figure 1(c)).

In order to validate the specificity of TAK-875 for inflammatory inhibition, we designed a specific agonist of TLR4, LPS, to induce inflammatory damage in *β*-cells and then intervened with TAK-875. TAK-875 attenuated the LPS-induced *β*-cell inflammatory apoptosis (Figure 1(d)), increased the insulin secretion (Figure 1(e)) and GPR40 expression, and inhibited TLR4 and NF-*κ*B subunit P65 expression (Figure 1(f)) in a concentration-dependent manner.

### 3.2. TLR4-NF-*κ*B Is Involved in the Protective Effect of TAK-875 on Lipotoxicity

**Figure 2:**
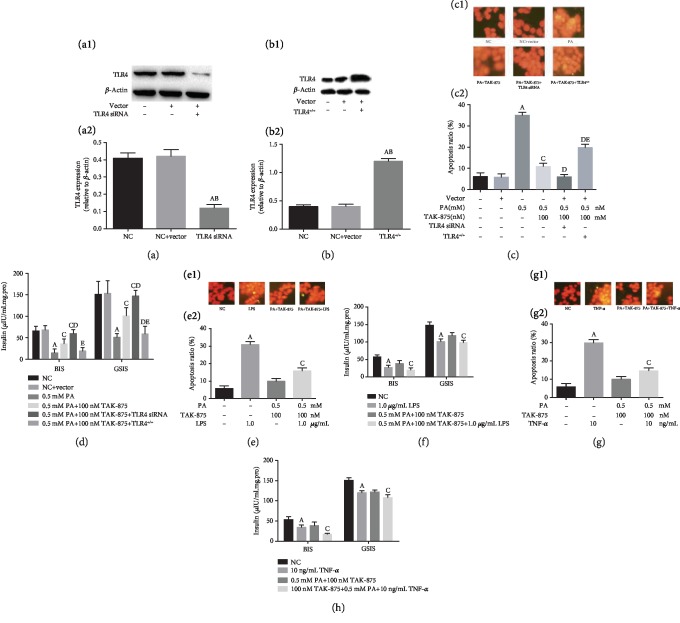
TLR4-NF-*κ*B is involved in the protective effect of TAK-875 on lipotoxicity. (a, b) TLR4 protein expression was detected by western blotting in TLR4-overexpressing and siRNA-transfected *β*-cells. (c, d) Regulation of TLR4 expression altered the protective effect of TAK-875 on PA-induced *β*-cells apoptosis (c), and insulin secretion disorder (d). ^a^*P* < 0.05 vs. NC group, ^b^*P* < 0.05 vs. NC+vector group, ^c^*P* < 0.05 vs. 0.5 mmol/L PA group, ^d^*P* < 0.05 vs. 0.5 mmol/L PA+100 nmol/L TAK-875 group, and ^e^*P* < 0.05 vs. 0.5 mmol/L PA+100 nmol/L TAK-875+TLR4 siRNA group. (e, f) Activation of TLR4 activity decreased the effect of TAK-875 on inhibition of PA-induced apoptosis (e) and insulin secretion disorder (f). (g, h) Activation of NF-*κ*B activity decreased the effect of TAK-875 on inhibited PA-induced apoptosis (g) and insulin secretion disorder (h). ^a^*P* < 0.05 vs. NC group and ^c^*P* < 0.05 vs. 0.5 mmol/L PA+100 nmol/L TAK-875 group.

We applied lentivirus-mediated *β*-cell TLR4 silencing (Figure 2(a)) or overexpression (Figure 2(b)) to verify whether TLR4 is involved in the protection of TAK-875. The results showed that increased TLR4 expression attenuated the benign intervention of TAK-875 on lipotoxic *β*-cell apoptosis (Figure 2(c)) and insulin secretion (Figure 2(d)), while the inhibition of TLR4 increased the above effect of TAK-875 on lipotoxic *β*-cells (Figures 2(c) and 2(d)).

Furthermore, to demonstrate the role of TLR4-NF-*κ*B signaling in TAK-875 intervention in lipotoxic-damaged *β*-cells, we further applied TLR4 and NF-*κ*B agonists that interfere with the effects of TAK-875 on lipotoxicity-injured *β*-cells. The results showed that the activation of TLR4 and NF-*κ*B attenuated the protective effect of TAK-875 on lipotoxicity-caused *β*-cell apoptosis (Figures 2(e) and 2(g)) and insulin secretion disorder (Figures 2(f) and 2(h)).

### 3.3. The Protective Effect of TAK-875 on Lipotoxicity Was Partially Independent of GPR40

**Figure 3:**
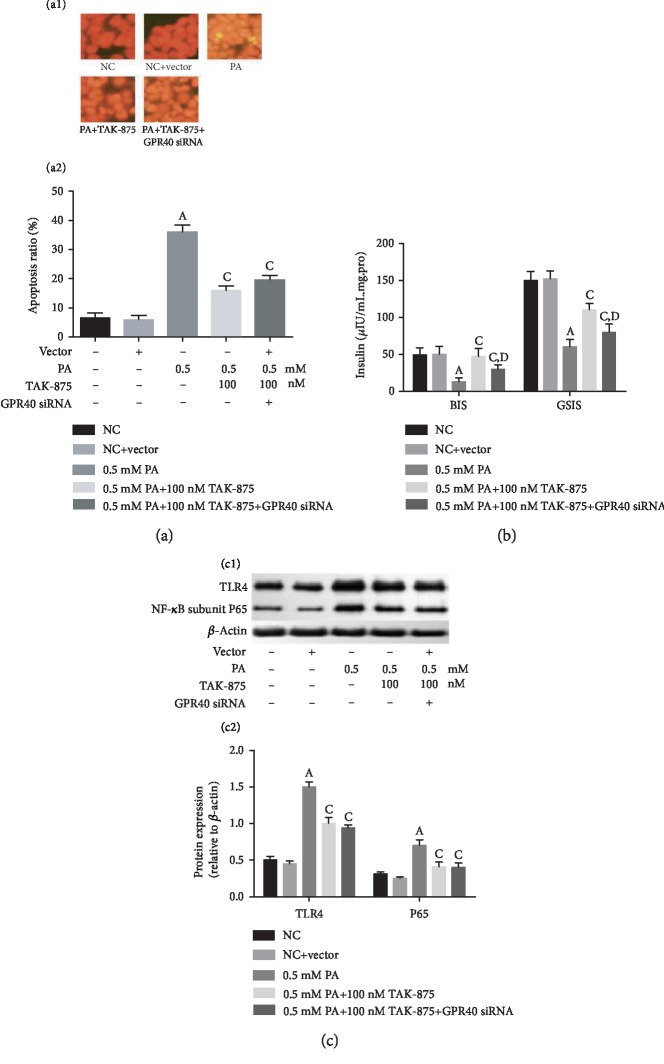
The protective effect of TAK-875 on lipotoxicity was partially independent of GPR40. (a) The effect of down regulation of GPR40 on the TAK-875 in inhibiting PA-induced apoptosis, (b) improving BIS and GSIS, and (c) decreasing inflammation-related protein expression. ^a^*P* < 0.05 vs. NC group, ^c^*P* < 0.05 vs. 0.5 mmol/L PA group, and ^d^*P* < 0.05 vs. 0.5 mmol/L PA+100 nmol/L TAK-875 group.

Furthermore, we demonstrated that TAK-875 functioned independent of GPR40 by using silencing of GPR40 in *β*-cells. Our results showed that downregulation of GPR40 had no significant effect on TAK-875 antagonizing PA-induced *β*-cell apoptosis (Figure 3(a)) and TLR4 and NF-*κ*B subunit P65 expression (Figure 3(c)) but inhibited the effect of TAK-875 on increasing BIS and GSIS (Figure 3(b)).

### 3.4. Effect of TAK-875 on HFD-Induced Metabolic Inflammation in Obese and TLR4^KO^ Rats

**Figure 4:**
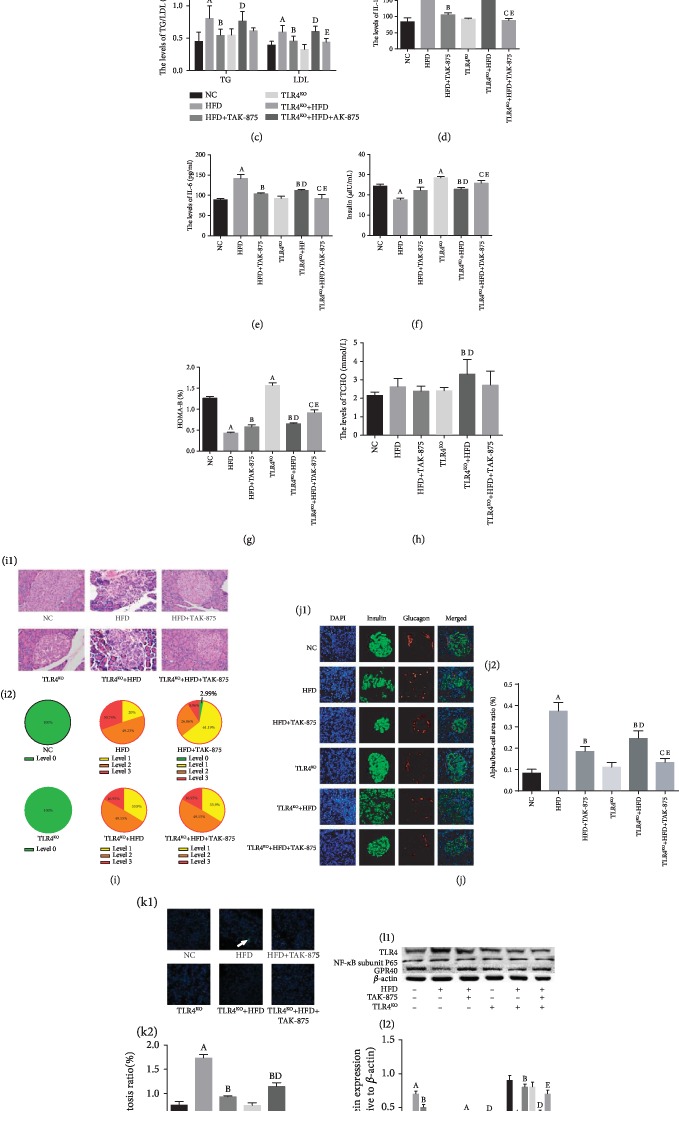
Protective effects of TAK-875 on pancreatic cells in obese rats and TLR4^KO^ rats is in synergy with TAK-875. (a) Average weight in each group. (b) The levels of fasting blood glucose. (c) The levels of blood lipid of each group: triglyceride (TG) and low-density lipoprotein (LDL). (d, e) The levels of blood inflammatory cytokines, IL-1 (d) and IL-6 (e). (f) Insulin levels. (g) HOMA-*β*. (h) The levels of total cholesterol (TCHO). (i) HE staining in pancreatic tissue. (j) The immunofluorescence images of *α* and *β*-cells in pancreatic tissues. (k) Apoptosis in pancreatic tissue (the white arrow indicates the apoptotic cells). (l) Expression of TLR4, NF-*κ*B, and GPR40 in pancreatic tissue. ^a^*P* < 0.05 vs. NC group, ^b^*P* < 0.05 vs. HFD group, ^c^*P* < 0.05 vs. HFD+TAK-875 group, ^d^*P* < 0.05 vs. TLR4^KO^ group, and ^e^*P* < 0.05 vs. TLR4^KO^+HFD group.

In order to verify the results of the current in vitro experiments, we investigated the role of TAK-875 in lipotoxic inflammatory injury on pancreatic cells of HFD-induced obese rats. The results revealed that TAK-875 reduced the body weight (Figure 4(a)), the fasting blood glucose (Figure 4(b)), and the levels of TG, LDL, IL-1, and IL-6 (Figures 4(c)–4(e)) but increased insulin levels (Figure 4(f)) and HOMA-B (Figure 4(g)) in HFD rats. There was no significant effects on the levels of TCHO (Figure 4(h)). TAK-875 improved the inflammatory infiltration of the pancreas (Figure 4(i)), improved the distribution of *α* and *β*-cells (Figure 4(j)), decreased the pancreatic cell apoptosis (Figure 4(k)), and expressed TLR4 and NF-*κ*B subunit P65 proteins (Figure 4(l)).

To investigate the role of TLR4 in chronic low-grade inflammation induced by HFD, we knocked out TLR4 in rats. Compared to the wild-type rats with HFD, TLR4^KO^ attenuated the weight gain induced by HFD (Figure 4(a)), improved *β*-cell function (Figures 4(f) and 4(g)) and the inflammatory infiltration (Figure 4(i)), and inhibited apoptosis (Figure 4(k)) and the expression of TLR4 and NF-*κ*B protein expression (Figure 4L). Similarly, TAK-875 intervention in the TLR4^KO^ obese group had lower inflammation (Figure 4(i)), pancreatic cell apoptosis (Figure 4(k)), and expression of TLR4 and NF-*κ*B subunit P65 proteins (Figure 4(l)) and better islet function (Figures 4(f) and 4(g)) as compared to TAK-875 intervention in the wild-type obese group.

## 4. Discussion

In this study, we used lipotoxic *β*-cells, obese SD rats, and a TLR4 knockout rat model to observe the role and mechanism of TAK-875 in lipotoxicity-induced injury in *β*-cells. The results showed that TAK-875 ameliorated PA-induced injury in islet *β*-cells and expression of TLR4-NF-*κ*B subunit P65 in a concentration-dependent manner. Moreover, silencing or overexpressing the TLR4 expression altered the protective effect of TAK-875 on lipotoxicity-damaged *β*-cells. Similarly, the activation of TLR4 or NF-*κ*B adjusted the effect of TAK-875 against PA-induced damage. Furthermore, the above process of TAK-875 was partially independent of GPR40 expression. TAK-875 reduced the body weight and inflammatory factors, inhibited the insulin resistance, rebalanced the number and distribution of *α* or *β*-cells, and inhibited the islet cell apoptosis and of the expression of TLR4-NF-*κ*B subunit P65 of obese rats. Further knockout of the rat TLR4 gene delayed the damage mentioned above induced by HFD in synergy with the action of the GPR40 agonist.

The current study showed that TAK-875 exerted a protective effect on the lipotoxic inflammatory injury in *β*-cells; however, TAK-875 alone interferes with the normal *β*-cells and does not exhibit any effect on cell apoptosis and TLR4-NF-*κ*B. This phenomenon led to the hypothesis that cell apoptosis under normal physiological conditions due to the activation of TLR4-NF-*κ*B was extremely small, and the changes caused by TAK-875 intervention could not be detected. Simultaneously, under physiological conditions, TAK-875 intervention does not increase the secretion of insulin in *β*-cells. Accumulating evidence indicated that TAK-875 alone does not cause hypoglycemia in diabetic patients [32], which supported our findings. Presently, several studies have confirmed that GPR40 agonists exert a protective effect on the *β*-cell lipotoxic damage [33]. However, the mechanism underlying the GPR40 agonist-delayed lipid damage of *β*-cells needs further investigation.

Some studies have shown that GPR40 prevents palmitate-induced *β*-cell death [34]. In addition, GPR40 activation decreased the inflammation-induced apoptosis [16]. Herein, we confirmed that TAK-875 induces lipotoxicity-induced *β*-cell apoptosis by inhibiting the TLR4-NF-*κ*B activation. Accumulating evidence confirmed that GPR40 is associated with immune inflammation [35]. For example, mice lacking GPR40 induced inflammation and insulin resistance in the brain [36]. Reportedly, the GPR40 signaling pathway plays a major role in the inhibition of spinal cord nociception after inflammation or nerve injury [37], and GPR40 antagonizes the periodontal inflammation [38]. These phenomena were in agreement with the current experimental results.

Although GPR40 agonist inhibits inflammation and has been confirmed in the studies of other tissues [39], in addition, the *β*-cells with GPR40 agonist exhibit an antagonistic effect on inflammation-induced apoptosis [40]. However, there is no direct evidence that TAK-875 inhibits metabolic apoptosis of *β*-cells by inhibiting the TLR4. Furthermore, we confirmed that the protective effects of TAK-875 were partially independent of GPR40 expression, and TAK-875 improved the hyperlipidemia-induced metabolic inflammatory injury by inhibiting the metabolic inflammatory pathway TLR4 in vivo. This result was consistent with the other studies [25], although we observed that TAK-875 intervention in the TLR4^KO^ rat group was significantly lower than the TAK-875 intervention in the wild-type. However, in the TLR4^KO^ high-fat group TAK-875 intervention or not, the difference was small. Since TLR4 plays a critical role in the process, HFD-induced damage after TLR4 knockdown was significantly inhibited. Moreover, TAK-875 exerted the effect by suppressing TLR4; the two roles overlap each other. Therefore, TAK-875 intervention did not show significant statistical differences in rats in the background of TLR4^KO^. In addition, our results showed that TLR4 protein is still expressed in TLR4^KO^ rats, the reason may be due to the fact that the total length of TLR4 gene was 600 bp, and we only knocked out more than 100 bp of the gene. The protein framework of TLR4 is present, and thus, the TLR4 protein can be detected by western blotting. Hence, we further applied PCR to detect TLR4 mRNA expression and found 436 bp of TLR4 in gene knockout rats, suggesting that the gene knockout was successful (see the attachment 1 for details).

The current study suggested that TAK-875 is a potential choice for obese prediabetes patients. Consecutively, TLR4 significantly inhibited the metabolic inflammation and oxidative stress induced by high lipotoxicity. Thus, developing TLR4 inhibitors is a potential weight loss target. Furthermore, our results suggested that the anti-inflammatory treatment in obese patients might prevent diabetes. Nevertheless, the present study had certain limitations. Firstly, we did not investigate the mechanism of TAK-875 acting on TLR4, and the direct or indirect effect between the two requires further research. Additionally, we used mouse islet cell line, which is different from islet *β*-cells found in humans; further research is warranted to confirm our experimental data.

## 5. Conclusion

In conclusion, we found that TAK-875 inhibits the lipotoxic inflammatory damage of *β*-cells through TLR4-NF-*κ*B signaling, and the above process of TAK-875 was partially independent of GPR40. This study revealed a new mechanism underlying the TAK-875-antagonized apoptosis in lipotoxic *β*-cells. Also, experimental evidence was provided for TLR4 as a therapeutic target for weight loss and intervention for *β*-cell metabolic damage.

## Data Availability

The data used to support the findings of this study are available from the corresponding authors upon request.
